# Silk Nanococoons: Bio‐Nanoreactors for Enzymatic Catalytic Reactions and Applications to Alcohol Intoxication

**DOI:** 10.1002/smsc.202000049

**Published:** 2021-02-10

**Authors:** Zhengwei Chen, Fan Hu, Zaifu Lin, Jin Hu, Runqing Shen, Youhui Lin, Xiang Yang Liu

**Affiliations:** ^1^ College of Ocean and Earth Sciences Research Institution for Biomimetics and Soft Matter Fujian Key Provincial Laboratory for Soft Functional Materials Research Xiamen University 422 Siming South Road Xiamen 361005 China; ^2^ Department of Physics National University of Singapore 2 Science Drive 3 Singapore 117542 Singapore; ^3^ State Key Laboratory of New Ceramics and Fine Processing School of Materials Science and Engineering Tsinghua University Beijing 100084 China

**Keywords:** alcohol intoxication, enzymatic catalysis, meso-functionalization, nanoreactors, silk fibroin

## Abstract

Enzymatic reactions are often terminated by severe aqueous environments. Inspired by bio‐protection of silk cocoons, a system of silk fibroin (SF) nanococoons is developed, which is capable of providing a bio‐protective environment for enzymatic activities on one hand, and facilitating the process of cascade enzymatic reactions on the other hand. This gives rise to the significant enhancement in enzymatic stability and efficacy. For instance, the protected glucose oxidase and horseradish peroxidase enzymes exhibit a 17.5‐ and 7.0‐fold increase when stored at 60 °C (>50% activity; ≈28 h) and 25 °C (>67.5% activity; ≈14 days), respectively, compared with free enzymes. It follows that this protective effect is subject to the mutual templated crystallization between enzymes and SF molecules, which prevents enzyme molecules from unfolding. In animal experiments, it shows that alcohol oxidase and catalase protected by silk nanococoons are able to reduce the alcohol levels of intoxicated mice with >5.0‐time efficacy at the alcohol decomposition level of >80% in 4.0 h compared with the unprotected. By bio‐inspiration of silk cocoons, the silk bio‐nanoreactors demonstrate the significant potential applications in biomedicine.

## Graphical abstract

The design of silk fibroin (SF) nanococoons provides a novel platform in protecting enzymatic activities with significant enhancements in enzymatic stability. The SF nanococoons functionalized with alcohol oxidase and catalase are shown to effectively reduce the alcohol levels of intoxicated mice. This gives rise to a novel platform for the design of advanced enzymatic biomaterials/biomedicine.

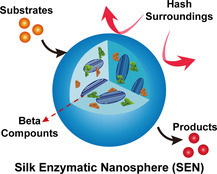

## 1 Introduction

Enzymatic catalysis is important in most life processes, and it is extensively applied in several research fields such as life sciences,^[^ ^1^ ^]^ environmental monitoring,^[^ ^2^ ^]^ biofuel production,^[^ ^3^ ^]^ and pharmaceutical research.^[^ ^4^ ^]^ However, free enzymes are extremely fragile in aqueous environments. Compared with the free‐enzyme systems, immobilized‐enzyme systems often show enhanced catalytic activity, thermal stability, and increased resistance to organic solvents.^[^ ^5^, ^6^ ^]^ Natural biomaterials are preferably adopted for the immobilization of enzymes due to their high biocompatibility and biodegradability.^[^ ^7^ ^]^ Natural silkworm cocoons are constructed by silkworms to protect the encapsulated pupae against predator attacks and environmental threats.^[^ ^8^ ^]^ Due to their unique hierarchical mesoscopic structures, cocoon silk materials exhibit outstanding mechanical properties, excellent biocompatibility, and controlled biodegradability.^[^ ^9^, ^10^ ^]^ In silk fibroin (SF) materials, mesoscopic reconstruction strategies include the doping of mesoscopic functional seeds or templates such as polymers,^[^ ^11^ ^]^ metal clusters,^[^ ^12^ ^]^ and carbon nanomaterials,^[^ ^13^ ^]^ to trigger the formation of SF β‐crystallite to reconstruct the mesoscopic structures and networks. This strategy endows SF materials with new functionalities, and is promising for application in the immobilization of multi‐enzymes for enhanced enzymatic catalytic performance. Herein, we demonstrated a type of silk enzymatic nanosphere (SEN) with stable microenvironments within which incompatible cascade reactions were conducted. This was achieved by immobilizing enzymes in SF materials, followed by meso‐functionalizing SF mesoscopic fibril networks. Our study provides a new strategy for constructing mesoscopic enzymatic catalysis systems and offers a novel platform for redesigning advanced biomaterials.

## 2 Results

### 2.1 Fabrication of SENs

**Figure 1:**
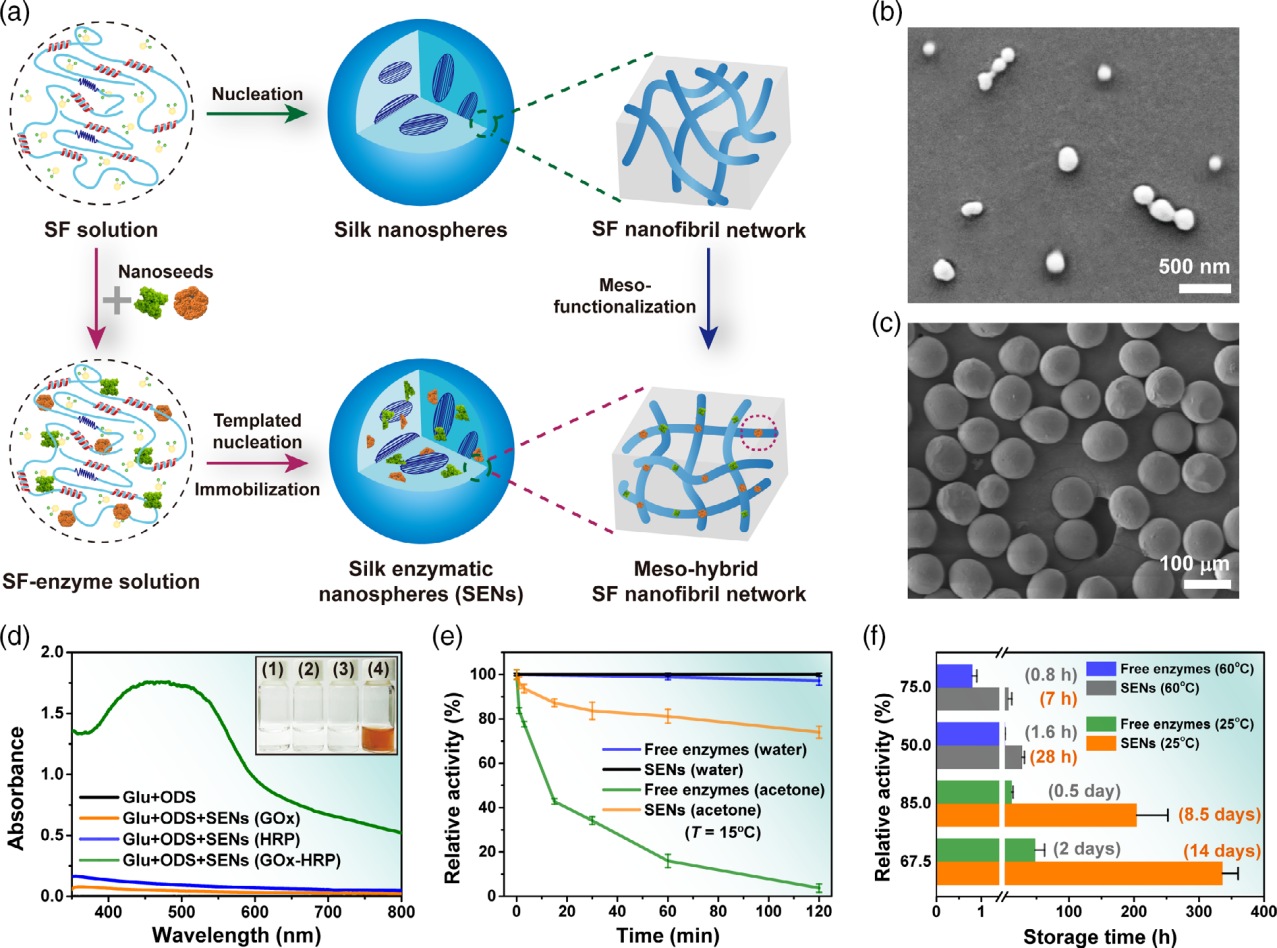
Enhanced activity and stability of SENs. a) Schematic illustration of the fabrication process of SENs. In SF mesoscopic hybrid material construction, the enzymes served as nanoseeds to incorporate them into the nanofibril networks of SF materials. b,c) Scanning electron microscopy (SEM) images of fabricated silk particles with size range of 0.03−100 μm; scale bars are 500 nm and 100 μm, respectively. d) The oxidations of ODS were recorded with a microplate reader: 1) only glucose; 2) glucose and SENs containing GOx; 3) glucose and SENs containing HRP; 4) glucose and SENs containing both GOx and HRP. e) Relative activities of 1) free enzyme (HRP) and 2) SENs (HRP/SF, with mass ratio: 1/120) after 2 h incubation in water at 15 °C; 3) free enzyme (HRP), and 4) SENs (HRP/SF, with mass ratio: 1/120) after 2 h incubation in acetone at 15 °C. f) Comparison of relative activities of the free enzymes and SENs incubated in aqueous solution at 60 °C and at 25 °C.

Cocoon silk from *Bombyx mori* silkworm consists of two main proteins: fibroin and sericin.^[^ ^10^ ^]^ Fibroin proteins are structural, whereas sericin proteins act as “glue‐like” factors that bind two fibroin fibers together.^[^ ^10^, ^14^ ^]^ We obtained regenerated SF solutions by removing sericin from cocoons and subsequently dissolving the fibroin fibers in protein denaturants (Scheme S1, Supporting Information).^[^ ^10^, ^14^, ^15^ ^]^ Furthermore, SENs were prepared from SF using a two‐step process: 1) mixing the regenerated SF solutions with selected enzymes, such as glucose oxidase (GOx) and horseradish peroxidase (HRP), and 2) generating SENs and simultaneously immobilizing enzymes into silk nanococoons following a mesoscopic functionalization strategy (**Figure** **1**a). In addition, adjusting the coagulant resulted in size‐adjustable SF spheres with diameter ranging from one hundred nanometer to hundreds of micrometer (Figure 1b,c). Note that SF nanospheres (with diameter of 30–250 nm) were used in this work (Figure S1, Supporting Information).

### 2.2 Enzyme Encapsulation of SENs

The meso‐reconstruction of SENs and their catalytic activity performance were demonstrated using GOx and HRP as model enzymes. Note that the enzyme activities of free enzymes (GOx/HRP) and the fabricated SENs (SF‐GOx/HRP nanospheres) were assessed by using glucose as substrates and monitoring the oxidation rate of *o*‐dianisidine (ODS) or 3,3′,5,5′‐tetramethylbenzidine (TMB; Figure S2, Supporting Information). Briefly, GOx catalyzes the oxidation of glucose and produces gluconic acid with hydrogen peroxide (H_2_O_2_), a toxic intermediate of this cascade reaction. Subsequently, the ODS or TMB gets oxidized by H_2_O_2_ under the catalysis of HRP, and this cascade reaction can be recorded with a plate reader in the absorbance mode (Supporting Information).

The kinetic analysis of the GOx–HRP reaction in SENs was investigated and compared with that of the free‐floating GOX/HRP mixture. The initial reaction rate of GOX–HRP versus glucose concentration followed typical Michaelis–Menten behavior. The Lineweaver–Burk plots (Figure S3 and Table S1, Supporting Information) show that the GOx–HRP system in SENs displayed a slightly higher Michaelis–Menten constant ($K_{M}^{\text{app}}$ = 53.95 × 10^−3^ m) to that of free GOX and HRP enzymes ($K_{M}^{\text{app}}$ = 37.76 × 10^−3^ m), indicating their comparable affinity to that of glucose.^[^ ^6^ ^]^ Figure 1d shows that the cascade reaction discussed earlier can be catalyzed by SENs. Alternatively, SENs exhibit the enzyme activities of both GOx and HRP.

To achieve higher enzyme encapsulation efficiency, a series of H_2_O_2_ conversion experiments catalyzed by changing molar ratios of HRP to SF from 1/30 to 1/960 were conducted in the SENs system, as the activity recovery of enzyme HRP in SENs was monitored by the oxidation of TMB. Figure S4a, Supporting Information, reveals that the increase in SF/enzyme content fraction in SENs significantly improved the recovery of enzyme activities (with identical enzyme amounts retained). Two factors can be attributed to this: 1) a higher SF content gives rise to higher enzyme encapsulation efficiency of SENs; 2) free enzymes (such as HRP) lose their activities quickly in acetone during the fabrication process. The SF nanofibril network of SENs “freezes” the protein configuration to protect enzymes from inactivation. A higher mass ratio of SF/enzymes leads to better protection. By adjusting the molar ratios of GOx to HRP from 1:9 to 49:1, the catalytic activity in the cascade reaction in the GOx–HRP system can be further optimized (Figure S4b, Supporting Information).

### 2.3 Stability of SENs

The enzymes meso‐functionalized in SENs displayed excellent stability under harsh conditions. For example, a significant enhancement of the stability of enzymes in the polarity organic solvent acetone was observed, as shown in Figure 1e. In the SEN system, >70% of activity was retained after 2 h of incubation in acetone (at 15 °C), whereas in the free‐enzyme system, only ≈3% of activity was retained under the same conditions. The higher SF content fraction provided better protection for enzymes in the SEN system (Figure S5, Supporting Information), similar to the protection of pupae provided by silk cocoons. The thermal stabilities of the enzymes in SENs were tested at 60 °C for 30 h (Figure 1f and Figure S6a, Supporting Information). After thermal treatment for ≈1.6 h at 60 °C, the free GOx lost half of its activity. After another 4 h of incubation, the free GOx was almost completely inactivated. In comparison with the free enzyme system, the SEN system retained ≈90% of its activity after ≈1.6 h of thermal treatment (Figure 1f and Figure S6a, Supporting Information). Notably, even after ≈28 h of thermal treatment, the SEN system retained >50% of the activity. As discussed earlier, the SENs contain high fractions of β‐crystallites comparable with those of natural silk cocoons, which exhibit high stabilities.^[^ ^10^ ^]^ Thus, the meso‐functionalization process of SENs affords enhanced enzyme stability.

Moreover, the enzymes meso‐functionalized in SENs displayed an excellent ability for long‐term storage at room temperature (25 °C). In general, natural enzymes lose their activities at room temperature, especially in aqueous solutions easily. In this regard, it takes quite a bit of cost to store and transport enzymes. For instance, as shown in Figure 1f and Figure S6b, Supporting Information, the free HRP aqueous solution retained ≈85% and ≈67.5% of activity after 0.5 and 2 days stored at 25 °C, respectively. It lost all activities after 2‐week storage at 25 °C. In contrast, the SENs were meso‐functionalized with the same amount of HRP; however, their storage stabilities were enhanced. Notably, the SEN system retained ≈85% of activity after being stored at 25 °C for 8.5 days (17 times compared with that of free enzymes) and retained ≈67.5% of activity after being stored in aqueous solution at 25 °C for 2 weeks (seven times compared with that of free enzymes), indicating a significant enhancement in the storage stability of enzymes at room temperature. The excellent storage stability of SENs not only certified their effective protection for enzymes but also provided a new method that enabled the reduction in the cost of storage and transportation of enzymes.

### 2.4 Protective Microenvironment for Cascade Reactions

**Figure 2:**
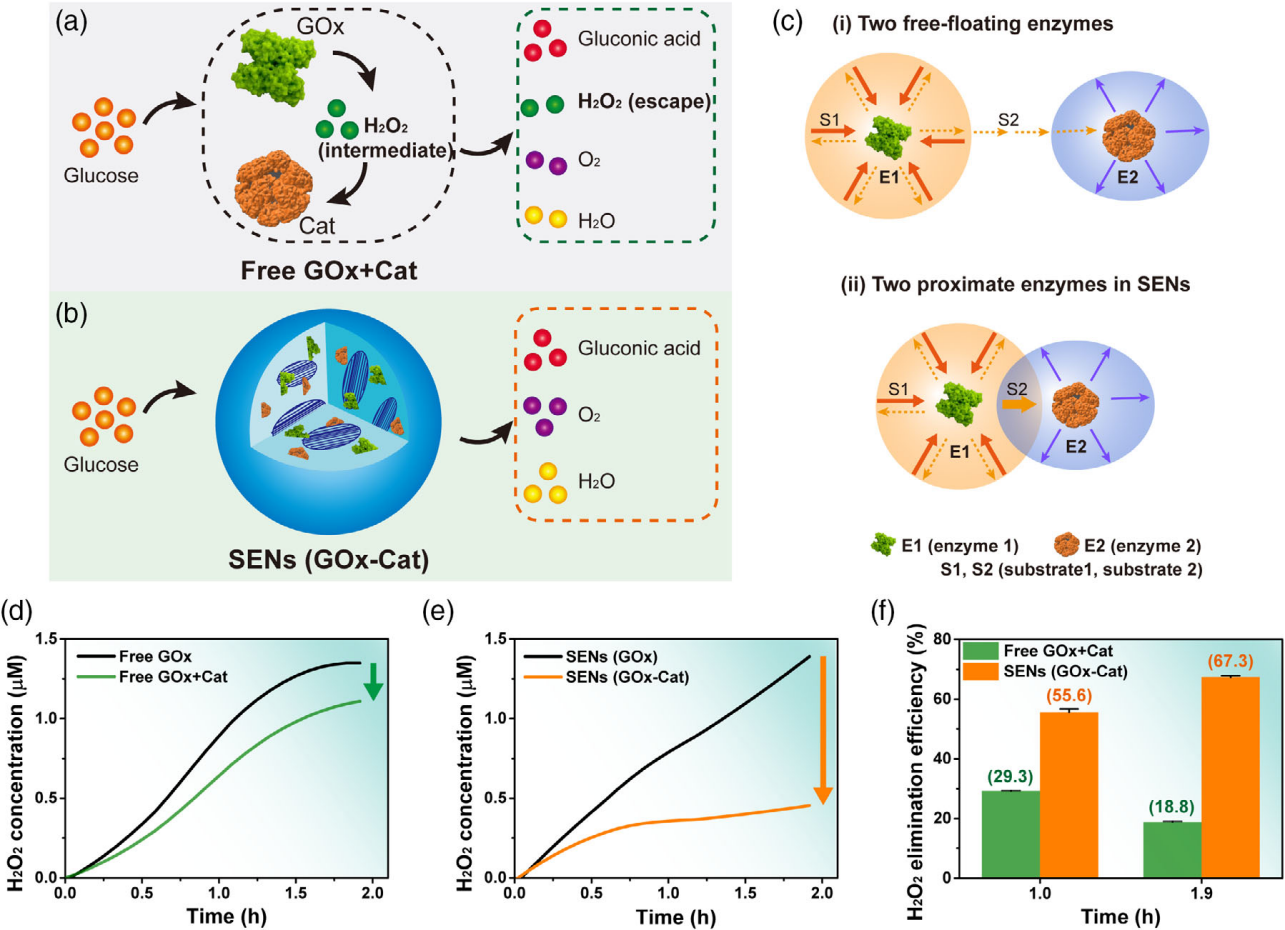
Enhanced H_2_O_2_ elimination efficiency of SENs. Illustration of the cascade reactions catalyzed by a) free GOx/Cat and b) SENs (SF–GOx/Cat nanospheres). c) Schematic illustration of the substrate channeling effect between i) two free‐floating enzymes (E1 and E2) and ii) two proximate enzymes (E1–E2). The diffusion of the substrates (S1 and S2) between enzymes (E1 and E2) is assumed to be directional. d,e) Production of H_2_O_2_ and f) calculated H_2_O_2_ elimination efficiency under the catalysis of free enzymes (GOx or GOx/Cat mixture) and SENs (containing GOx or a combination of GOx and Cat; Supporting Information). The standard absorbance curve at various H_2_O_2_ concentration is shown in Figure S8, Supporting Information.

Similar to the protection function of silk cocoons, the meso‐functionalized SEN system provided a favorable environment for enzymatic catalytic reactions, especially for the cascade reactions of multiple enzymes. We conducted a group of glucose conversion experiments using free enzymes and the SEN system, respectively (**Figure** **2**a,b). The efficiencies of eliminating H_2_O_2_ were calculated by the concentration changes. As shown in Figure 2c, compared with 1) two free‐floating enzymes (E1 and E2) in the solution state, the SEN system exhibited higher catalytic activity due to the faster substrate channeling between 2) two proximate enzymes (E1–E2) meso‐functionalized in SF networks.^[^ ^16^ ^]^ In the meso‐reconstructed hierarchical SENs, GOx and HRP were homo‐dispersed and closely packaged, facilitating H_2_O_2_ transfer among enzymes (also called the proximity effect) and minimizing H_2_O_2_ leakage.^[^ ^17^ ^]^

As shown in Figure 2d−f, the SEN (GOx–Cat) system exhibited a 3.6‐fold increase in H_2_O_2_ eliminating efficiency compared with that of the free enzyme system (with identical enzyme amounts). More than 67% of produced H_2_O_2_ was decomposed by the SEN system for 1.9 h. Notably, the H_2_O_2_ eliminating efficiency of the free enzyme system was decreased from ≈29.3% (in 1.0 h) to ≈18.8% (in 1.9 h), suggesting a strong inhibitory effect of a high local H_2_O_2_ concentration on Cat activity in the free enzyme system. In contrast, the SEN system maintained a high efficiency of eliminating H_2_O_2_ (≈55.6% in 1.0 h and ≈67.3% in 1.9 h). Benefiting from the closely packaged microenvironment (Figure 2c), on one hand, intermediates were efficiently transferred among enzymes, promoting the elimination of toxic intermediates (such as H_2_O_2_); on the other hand, adjusting the distance between GOx and Cat by the reconstructed SF mesoscopic structures afforded higher Cat activity by minimizing the H_2_O_2_ inhibitory effect,^[^ ^18^ ^]^ sustainably and efficiently triggering the cascade reaction. Moreover, even under high‐viscous‐liquid environments (similar to those within cells or blood),^[^ ^19^ ^]^ the SEN system retained a threefold higher catalytic activity than that of the free enzyme system in 20 v/v% poly(ethylene glycol) (PEG) solution (Figure S7, Supporting Information). Consequently, the SEN system provided a stable microenvironment for cascade reactions, which promoted the elimination of toxic intermediates and enhanced its ability in highly viscous environments.

### 2.5 Bio‐Nano‐Antidotes for Alcohol Intoxication

**Figure 3:**
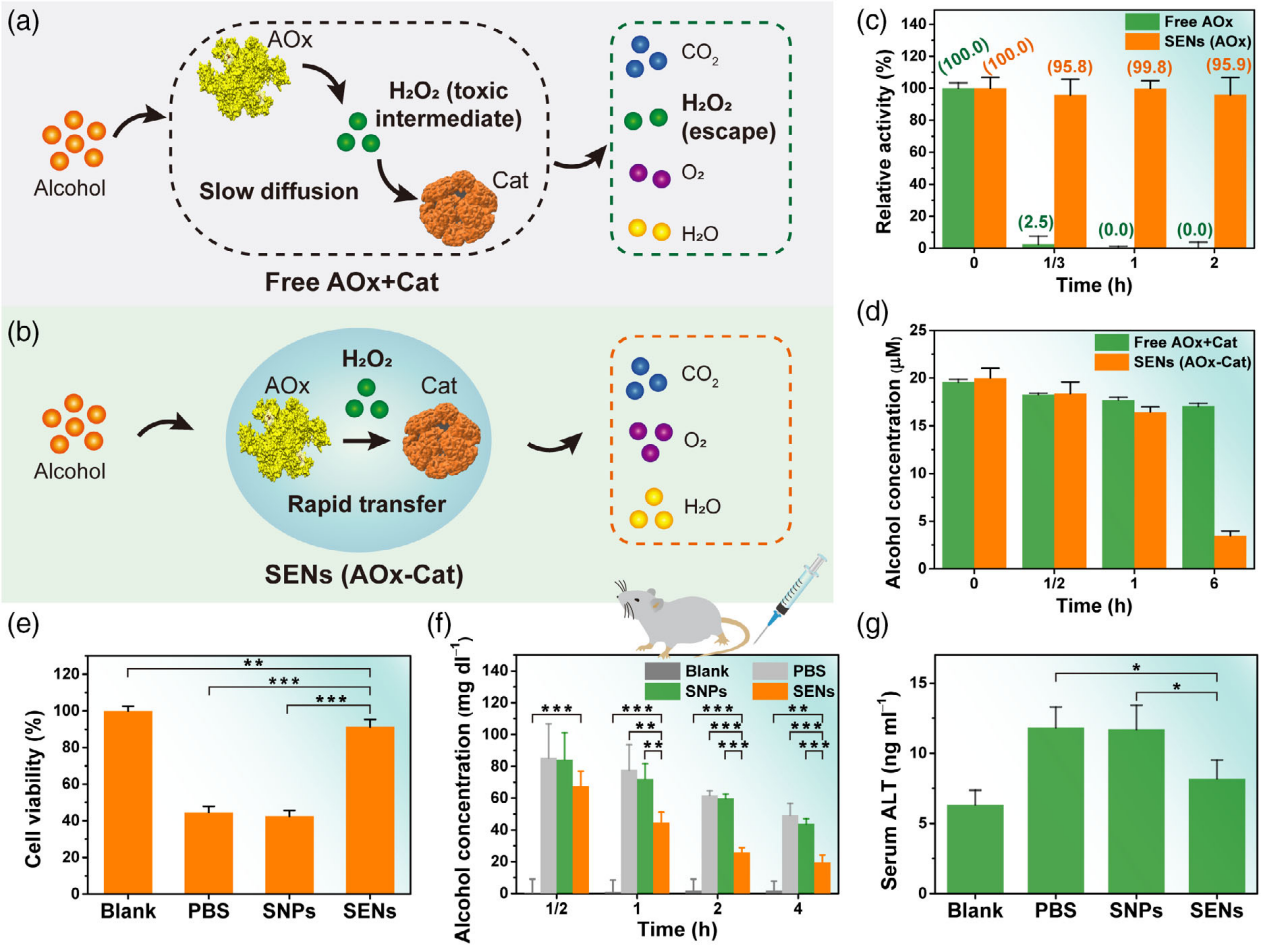
Efficacy of SENs as bio‐nano‐antidotes for alcohol intoxication. Illustration of the cascade reactions catalyzed by a) free AOx/Cat and b) SENs (SF–AOx/Cat nanospheres). c) Relative activities of free enzymes (AOx) and SENs containing identical enzyme amounts incubated in the simulated intestinal fluid (pH = 7.6, 0.1 m phosphate buffer, containing 1 × 10^−5^ m trypsin) at 37 °C. d) Residuals of alcohol in the simulated intestinal fluid (with the addition of alcohol (2 × 10^−5^ m) at 37 °C) after catalysis reaction of free enzymes (AOx/Cat, enzyme molar ratio: 1) or SENs containing identical enzyme amounts. e) Cell viability assays after treatment with PBS, SNPs, or SENs (SF–AOx/Cat nanospheres) together with ethanol (final concentration 4%) for 4 h at 37 °C. Cell viability rates were normalized with those of the untreated cells (blank group). f) BAC and g) ALT in mice after gavage with an alcohol diet (2 mg ethanol per gram body weight) containing PBS, SNPs, or SENs (SF–AOx/Cat nanospheres). The volumes of PBS and enzyme solutions injected were maintained at 300 μL. The dose of enzyme injected was maintained at 60 μg AOx or 21 μg Cat per mouse. Blank group of mice was fed with an isocaloric diet without ethanol and was used as the baseline group. Four mice were used in each group. The significance levels are *P < 0.05, **P < 0.01, and ***P < 0.001. *P* < 0.05 or less was considered significant.

The meso‐functionalization strategy to afford SENs with enhanced enzymatic activity and stability offers a novel platform for numerous applications. For example, alcohol consumption is a traditional part of civilization over thousands of years, especially in China.^[^ ^20^ ^]^ Excessive consumption of alcohol has resulted in severe social problems and serious damage to human organs such as the brain and liver.^[^ ^21^ ^]^ Although medical advancements have been made to treat alcohol intoxication,^[^ ^22^ ^]^ they have limited efficiency and several side effects. In this regard, new medicines for alcohol intoxication are required. Herein, alcohol oxidase (AOx) and catalase (Cat) were meso‐functionalized in the SEN system to fabricate a multi‐enzyme system as an antidote for alcohol intoxication. Compared with the performance of the free enzyme system, the SENs (SF–AOx/Cat nanospheres) effectively eliminated both alcohol and toxic intermediates H_2_O_2_ (**Figure** **3**a,b). Moreover, due to the exceptional biocompatibility of SF, the SENs may avoid causing inflammatory and immunogenic responses in human bodies.^[^ ^9^, ^10^, ^11^ ^]^ A test of the biological ability of the SEN system against protease was conducted in an AOx–Cat system using trypsin as a model digesting enzyme (Figure 3c and Figure S9a, Supporting Information). The free AOx system lost ≈97.5% of activity after trypsin digestion for 20 min, whereas the SENs (with the same amount of AOx) exhibited extraordinary protease resistance, retaining >95% of activity after the same digestion treatment for 2 h. This makes oral administration of SEN‐based medicines feasible.

To investigate the efficacy of alcohol intoxication in simulated biological environment, free AOx/Cat enzymes and SENs (with identical amounts of AOx/Cat) were placed in the simulated intestinal fluid (at 37 °C) with the addition of alcohol (2 × 10^−5^ m), respectively. As shown in Figure 3d and Figure S9b, Supporting Information, the free enzyme system lost most of its ability to decompose alcohol after only 1 h of incubation, likely due to the hydrolysis of free enzymes by proteases. In contrast, the SEN system maintained its high efficacy of alcohol intoxication and eliminated >80% of alcohol in 6 h. Moreover, the efficacy of alcohol intoxication was evaluated using cell viability assays (Figure 3e and Figure S10, Supporting Information). Note that the pure silk nanoparticles (SNPs) exhibited good cell compatibility (with ≈100% of cell viability in 0−80 μg mL^−1^ SNP solution for 4 h; Figure S10a, Supporting Information). The addition of ethanol dramatically decreased cell viability (lost >50% of cell viability at 4% ethanol) with no antidotes (Figure S10b, Supporting Information). Cells incubated with phosphate buffer saline (PBS) and SNPs in 4%‐alcohol‐containing media lost >55% of their viability, whereas the addition of SENs (AOx–Cat) into media increased the viability to ≈91% (Figure 3e), similar to that of the control (untreated cells). The meso‐functionalized SEN system protected cells from alcohol and H_2_O_2_ toxicity and maintained their viability in a simulated biological environment.

Subsequently, the efficacy of alcohol intoxication was further investigated by in vivo experiments (Figure 3f,g). PBS, SNPs, or SENs (SF–AOx/Cat) were mixed in an alcoholic diet (with 2 mg ethanol per gram body weight) and then gavaged to male C57B6 mice (8 weeks old). As shown in Figure 3f, the blood alcohol concentration (BAC) of mice gavaged with SENs was apparently smaller than that of the others, indicating good efficacy of SENs as an antidote for alcohol intoxication. In addition, all alcohol‐fed animals showed increased plasma alanine aminotransferase (ALT) levels, which signified the extent of damage to the liver.^[^ ^23^ ^]^ However, the mice treated with SENs had the lowest ALT levels except the blank group (Figure 3g). Our findings demonstrated that the SENs exhibited good efficacy as antidotes for alcohol intoxication and exhibited promising applications in biomedicine.

### 2.6 Protective Mechanism of SENs

**Figure 4:**
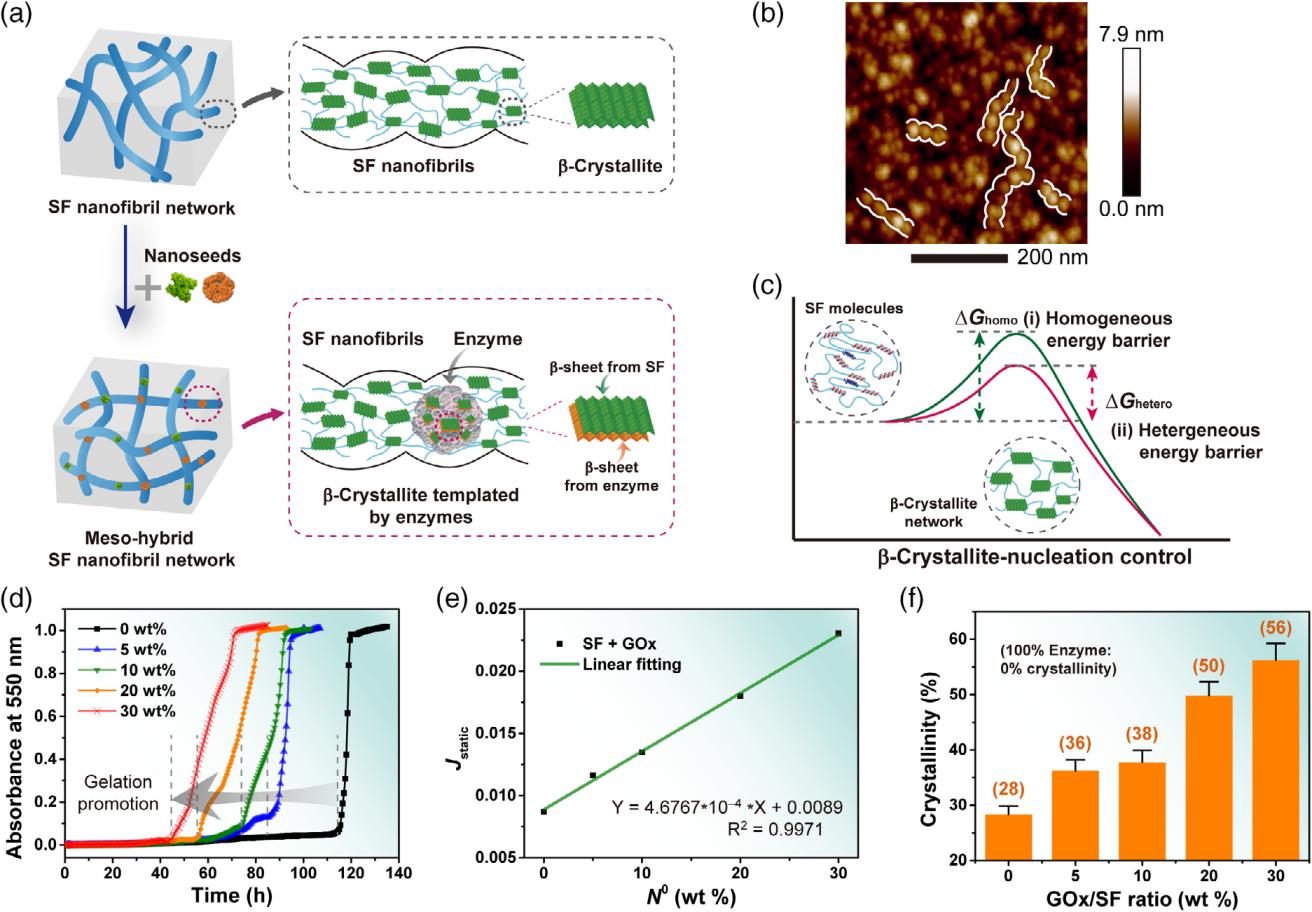
Protective mechanism of SENs. a) Schematic illustration of the protective mechanism of SENs. Enzymes were “frozen” in the SF nanofibril network in SENs. b) Atomic force microscopy (AFM) height image of an enzyme‐functionalized SF film. c) Schematic illustration of nucleation control for the formation of these structures. d,e) Normalized optical density (OD) changes in pure SF solution and SF–enzyme mixture. The SF concentration was 5 wt%, and the concentrations of enzymes named GOx were 0, 5, 10, 20, and 30 wt% compared with that of SF. *T* = 25 °C. Gelation time (*t*_g_) was defined as the time when the OD value sharply increased. GOx promoted β‐crystallization, as can be seen from the shortened gelation time (*t*_g_ ≈ 115, 89, 72, 55, and 45 h, respectively). f) Chart summarizing the crystallinity changes in GOx/SF samples at various GOx/SF ratios (0, 5, 10, 20, and 30 wt%; with the concentration of SF kept at 5 wt%, after ≈60 h of incubation at *T* = 25 °C), calculated from the XRD spectra (Figure S10, Supporting Information). Note that the crystallinity of pure enzymes was calculated ≈0%.

By immobilizing functional enzymes in SF nanofibril networks, the SEN system exhibited a great improvement in stability and catalytic efficiency compared with that of the free enzymes and was proven to be an alternative antidote for alcohol intoxication. The enhanced performance was attributed to the mutual intermolecular crystallization between enzymes and SF molecules, which may partially freeze molecular conformation of the enzymes to some degree in the meso‐functionalization process (**Figure** **4**a). Specifically, in the preparation of SENs, enzymes were added into SF solution as nanoseeds/templates, and acetone was a coagulant for gelation of SF solutions, contributing to the instantaneous harvesting of high‐crystalline SENs. The enzymes were submerged in the SF nanofibril matrices (Figure 4b). The meso‐functionalization process was referred to as template nucleation theory. According to the typical nucleation theory,^[^ ^24^, ^25^, ^26^ ^]^ nucleation is divided into homogeneous and heterogeneous nucleation, and the nucleation rate *J* in the SF nanofibril network formation is given by Equation (1) and (2).^[^ ^27^ ^]^

(1) $$J=A\text{exp}[-\Delta G^{*}f/kT]\times N^{0}$$

with

(2) $$\Delta G^{*}=B/{\left(\Delta \mu \right)}^{2}$$

where *A* and *B* are the kinetic constants, $\Delta G^{*}$ is the nucleation barrier, *f* is the interfacial correlation factor between the nucleating phase and templates (0 < *f* ≤ 1; for homogeneous nucleation, *f* = 1),^[^ ^9^, ^26^, ^27^ ^]^ *k* is Boltzmann's constant, *T* is the ambient temperature, *N* ^0^ is the density of nucleating templates, and Δμ is the chemical potential difference between the ambient mother and crystalline phases.

The added enzymes (as nanoseeds) lowered the nucleation barrier to accelerate the nucleation process (Figure 4c),^[^ ^28^ ^]^ and the enzyme concentrations (*N* ^0^) directly promoted the nucleation rate (*J*). With the increase in enzyme addition, the gelation time (*t*_g_), which corresponds to the static induction time of nucleation of β‐crystallites and nanofibril/β‐crystallite networks, was shortened (Figure 4d). This implied that in SF heterogeneous nucleation, the protein nucleation barrier was significantly reduced if the foreign substances had strong interactions with the crystalline phase.^[^ ^27^ ^]^ Based on the fact that *J*_static_ ≈ 1/*t*_g_,^[^ ^25^, ^29^ ^]^ the static nucleation rate (*J*_static_) was proportional to the concentration of added enzymes (*N* ^0^), which means that *J*_static_ ≈ *N* ^0^ (Figure 4e). The results demonstrated that the added enzymes promote the nucleation of β‐crystallites and nanofibril/β‐crystallite networks of SF in SF/enzyme solutions, significantly decreasing the gelation time (*t*_g_) compared with that of a pure SF solution (with 0 wt% of enzymes added). The enzyme acted as nucleation templates to promote the nucleation/formation of SF materials. Subsequently, the SF mesoscopic structures probably “frozen” the enzymes structurally.

To further quantify the β‐crystallite contents, we calculated the crystallinity of enzymes/SF samples at different enzymes/SF ratios acquired from X‐ray diffraction (XRD) (Figure 4f and Figure S11, Supporting Information). A positive correlation was found between the crystallinities of the enzyme/SF samples and the concentrations of added enzymes. When 30 wt% of GOx (compared with that of SF) was introduced, the crystallinity of enzymes/SF significantly increased to 56%, similar to that of natural silk cocoons compared with 28% of the pure SF sample (with 0 wt% of GOx added). In this regard, meso‐hybrid SF materials achieved a high crystallinity comparable to that of silk cocoons with a shortened incubation time. As discussed earlier, free enzymes easily lose their activities in harsh environments, whereas SENs demonstrated significantly enhanced catalytic performance due to protection from SF mesoscopic structures.

## 3 Discussion

We demonstrated a type of nature‐inspired SEN design for stable cascade biocatalysis by “freezing” enzymes within mesoscopic SF nanofibril networks. Our SEN system exhibited remarkable advantages, such as the high recovery of enzyme activity and the adjustable ratio of multi‐enzymes. From mesoscopic structures to macroscopic properties, the “freezing” function of the meso‐reconstructed SF β‐crystallite/fibril networks gave rise to high enzyme encapsulation, which significantly enhanced thermal stabilities (stored for ≈28 h at 60 °C with >50% of activity retained; 17.5 times that of free enzymes) and storage stability (stored for 2 weeks at 25 °C with >67.5% of activity retained; 7 times that of free enzymes). Notably, SENs were capable of providing favorable microenvironments for enzymatic catalytic cascade reactions that promoted the elimination of toxic intermediates (with ≈67.3% of H_2_O_2_ elimination efficiency at 1.9 h; 3.6 times that of free enzymes) and also enhanced the activities under high‐viscous environments (with >60% of activity retained at 20% PEG solution for 4 h; 3 times that of free enzymes). Moreover, through meso‐functionalization with AOx and Cat, this targetable SEN design can be applied as a bio‐nano antidote for alcohol intoxication, as it exhibited excellent protease resistance (with >95% of activity retained in 2.0 h) and a remarkable efficacy of alcohol intoxication in intoxicated mice (with >80% alcohol decomposed and >5.0‐time efficacy compared with pure SF nanoparticles in ≈4.0 h), suggesting its promising application prospect in biomedicine.

By “freezing” functional enzymes in SF mesoscopic structures, functionalized SF materials exhibited enhanced stability and catalytic efficiency. The meso‐functionalization strategy was introduced to design and fabricate SF‐based nanomaterials, serving as a novel platform for numerous biomedical applications. Moreover, SF hierarchical mesoscopic structures are crucial for achieving macroscopic performance. The innovative strategy of engineering soft materials at the mesoscale demonstrates a novel route for redesigning and reconstructing advanced functional materials with extraordinary performance. Our findings highlight a bio‐mimicking silk‐based functional nanomaterial and offer an innovative strategy for mesoscopic engineering of soft materials.

## Conflict of Interest

The authors declare no conflict of interest.

## Author Contributions

All authors jointly conceptualized the article and contributed to the original and revised drafts.

## Supporting information

Supplementary Material

## References

[1] G. J. Williams , A. S. Nelson , A. Berry , Cell. Mol. Life Sci. 2004, 61, 3034. 15583865 10.1007/s00018-004-4234-5 PMC11924439

[2] S. K. Ahuja , G. M. Ferreira , A. R. Moreira , Crit. Rev. Biotechnol. 2004, 24, 125. 15493529 10.1080/07388550490493726

[3] M. Puri , C. J. Barrow , M. L. Verma , Trends Biotechnol. 2013, 31, 215. 23410582 10.1016/j.tibtech.2013.01.002

[4] J. M. Woodley , Trends Biotechnol. 2008, 26, 321. 18436317 10.1016/j.tibtech.2008.03.004

[5] a) Y. F. Zhang , J. Ge , Z. Liu , ACS Catal. 2015, 5, 4503;

[6] H. L. Tan , S. Guo , N. D. Dinh , R. C. Luo , L. Jin , C. H. Chen , Nat. Commun. 2017, 8, 663. 28939810 10.1038/s41467-017-00757-4 PMC5610232

[7] a) W. P. J. Appel , E. W. Meijer , P. Y. W. Dankers , Macromol. Biosci. 2011, 11, 1706; 21919208 10.1002/mabi.201100225

[8] H. P. Zhao , X. Q. Feng , S. W. Yu , W. Z. Cui , F. Z. Zou , Polymer 2005, 46, 9192.

[9] N. B. Lin , X. Y. Liu , Chem. Soc. Rev. 2015, 44, 7917. 26288339 10.1039/c5cs90084k

[10] W. Qiu , A. Patil , F. Hu , X. Y. Liu , Small 2019, 15, 1903948. 10.1002/smll.201903948 31657136

[11] J. Huang , Z. Xu , W. Qiu , F. Chen , Z. Meng , C. Hou , W. Guo , X. Y. Liu , Adv. Funct. Mater. 2020, 30, 1910547.

[12] C. Shi , J. Wang , M. L. Sushko , W. Qiu , X. Yan , X. Y. Liu , Adv. Funct. Mater. 2019, 29, 1904777.

[13] L. Ma , Q. Liu , R. Wu , Z. Meng , A. Patil , R. Yu , Y. Yang , S. Zhu , X. Fan , C. Hou , Y. Li , W. Qiu , L. Huang , J. Wang , N. Lin , Y. Wan , J. Hu , X. Y. Liu , Small 2020, 16, e2000203. 32452630 10.1002/smll.202000203

[14] F. Hu , N. B. Lin , X. Y. Liu , iScience 2020, 23, 101035. 32311584 10.1016/j.isci.2020.101035 PMC7168770

[15] F. Hu , W. Z. Liu , W. F. Li , Z. J. Xu , Y. Y. Diao , N. B. Lin , W. X. Guo , L. Shi , J. H. van Esch , X. Y. Liu , Small 2019, 15, 1804171. 10.1002/smll.201804171 30786154

[16] E. T. Hwang , S. Lee , ACS Catal. 2019, 9, 4402.

[17] Y. Liu , J. J. Du , M. Yan , M. Y. Lau , J. Hu , H. Han , O. O. Yang , S. Liang , W. Wei , H. Wang , J. M. Li , X. Y. Zhu , L. Q. Shi , W. Chen , C. Ji , Y. F. Lu , Nat. Nanotechnol. 2013, 8, 187. 23416793 10.1038/nnano.2012.264 PMC3670615

[18] O. I. Wilner , Y. Weizmann , R. Gill , O. Lioubashevski , R. Freeman , I. Willner , Nat. Nanotechnol. 2009, 4, 249. 19350036 10.1038/nnano.2009.50

[19] R. J. Ellis , Trends Biochem. Sci 2001, 26, 597. 11590012 10.1016/s0968-0004(01)01938-7

[20] J. Cochrane , H. H. Chen , K. M. Conigrave , W. Hao , Alcohol Alcohol 2003, 38, 537. 14633640 10.1093/alcalc/agg111

[21] J. Rehm , C. Mathers , S. Popova , M. Thavorncharoensap , Y. Teerawattananon , J. Patra , Lancet 2009, 373, 2223. 19560604 10.1016/S0140-6736(09)60746-7

[22] a) S. Martins , B. Sarmento , D. C. Ferreira , E. B. Souto , Int. J. Nanomed. 2007, 2, 595; PMC2676808 18203427

[23] R. Kwok , K. C. Choi , G. L. H. Wong , Y. Y. Zhang , H. L. Y. Chan , A. O. Y. Luk , S. S. T. Shu , A. W. H. Chan , M. W. Yeung , J. C. N. Chan , A. P. S. Kong , V. W. S. Wong , Gut 2016, 65, 1359. 25873639 10.1136/gutjnl-2015-309265

[24] a) Y. Y. Diao , X. Y. Liu , Adv. Funct. Mater. 2012, 22, 1354;

[25] K. Q. Zhang , X. Y. Liu , Nature 2004, 429, 739. 15201905 10.1038/nature02630

[26] Z. S. Zhang , X. Y. Liu , Chem. Soc. Rev. 2018, 47, 7116. 30137078 10.1039/c8cs00626a

[27] T. H. Zhang , X. Y. Liu , Chem. Soc. Rev. 2014, 43, 2324. 24435291 10.1039/c3cs60398a

[28] Z. W. Chen , H. H. Zhang , Z. F. Lin , Y. H. Lin , J. H. van Esch , X. Y. Liu , Adv. Funct. Mater. 2016, 26, 8978.

[29] X. Y. Liu , in Advances in Crystal Growth Research (Eds: K. Sato , Y. Furukawa , K. Nakajima ), Vol. 1, Elsevier, Amsterdam, The Netherlands 2001, Part 1.

