# Supplementary material for: Silk Nanococoons: Bio‐Nanoreactors for Enzymatic Catalytic Reactions and Applications to Alcohol Intoxication
Source: Small Sci. 2021 Feb 10;1(3):2000049. doi: 10.1002/smsc.202000049 (PMC11935913; doi:10.1002/smsc.202000049)
Supplement: Supplementary file 1 — Supplementary Material [file SMSC-1-2000049-s001.docx]

Copyright WILEY-VCH Verlag GmbH & Co. KGaA, 69469 Weinheim, Germany, 2018.

Supporting Information

**Using Silk Nanococoons as Enzymatic Nanoreactors to Treat Alcohol Intoxication**

*Fan Hu, Zhengwei Chen, Zaifu Lin, Jin Hu, Runqing Shen, Youhui Lin, and Xiang Yang Liu**

(F. Hu and Z. Chen contributed equally to this work.)

**Text S1: Methods**

**Materials.** *Bombyx mori* silkworm cocoons provided by Guangxi Sericulture Technology Co., Ltd (Guangxi, China). Horse radish peroxidase (HRP; >300 U mg^−1^), glucose oxidase (GOx; >100 U mg^−1^), catalase (Cat; >20 kU mg^−1^), alcohol oxidase (AOx; 14 U mg^−1^), trypsin (>10,000 BAEE units mg^−1^), o-Dianisidine (ODS) and 3,3′,5,5′-tetramthylbenzidine (TMB) were purchased from Sigma-Aldrich (Shanghai, China). Sodium carbonate (Na_2_CO_3_) and acetone were obtained from Xilong Chemical Industry. Lithium bromide (LiBr) were provided by Aladdin Industrial Corporation. Ethanol assay kit was purchased from BioVision (St. California, San Francisco, USA). Sodium bicarbonate, lithium bromide, dipotassium phosphate, monopotassium phosphate and glucose were purchase form Aladdin (Shanghai, China). Deionized (DI) water with a specific resistance of >18.2 MΩ cm^−1^ was used throughout the experiment

**Preparation of Silk Fibroin Solutions.** Silk fibroin (SF) proteins were extracted from *Bombyx mori* cocoons and degummed in boiling aqueous solution of 0.5% (w/w) Na_2_CO_3_ for 30 min twice with frequent stir. After that, the degummed silk fibers were washed with DI water at 60°C for 5 times. The regenerated SF solution was obtained by dissolving the degummed silks into 9.3 м LiBr solution for 8 h at 60°C, and then extracting LiBr from SF solution via a dialysis cassette (Solarbio, molecular weight cut-off 3500) for 2 days with frequent change of DI water (**Scheme S2**).

**Fabrication of Silk-Enzyme Nanoreactors.** The silk fibroin solution (6% w/w) was mixed with enzyme solutions at given mass ratios for various silk-enzyme nanoreactors (SENs; **Scheme S1**). The silk nanoparticles (SNPs) contained no enzymes. After more than 3 h homogenization at 4°C, the SF-enzyme solution was gently introduced into acetone through a sample injector (~150 μl drop^−1^), and make sure that the final volume ratio of acetone should be >75% (v/v). The SENs were immediately formed and suspended in the liquid comprised of water and acetone. Then the SENs were collected by centrifugations at 18,000 rpm and subsequently re-suspended in DI water by sonication and shaking. The centrifugation and re-suspension steps were repeated at least twice to purify the SEN sample. All the supernates after centrifugation were collected to detect the fraction of none entrapped enzymes. After the final spinning, the suspension was progressively filtered using 0.45-μm and 0.22-μm polyvinylidene fluoride (PVDF) filters. Then the suspension was frozen by liquid nitrogen, and then transfer to a freeze dryer (Scanvac coolsafe 110, LaboGene, Danmark) and lyophilized at −100°C for 3 days. The SEN powders were stored at 4ºC before further usage.

**Note S1. Fabrication process of SENs.**

At first, glucose oxidase (GOx) and horseradish peroxidase (HRP) were employed as model enzymes and mixed with SF solutions. After more than 3 h homogenization at 4°C, the SF-enzymes mixture was gently introduced into the acetone to generate SENs through a phase-separation procedure. Due to the interfacial tension, the SF peptide chains were inclined to form spherical shapes and wrapped enzymes inside. After several times of washing, pure SENs can be obtained (Figure 2).

**Morphology Characterization of SENs/SNPs.** 20-μl diluted SNPs or silk-enzyme nanoreactors (SENs) suspension was applied to a silica slide, frozen by liquid nitrogen and lyophilized at −100ºC for 1 day by a freeze-dryer. The size and morphology of SNPs or SENs was characterized by a scanning electronic microscope (SU-70, Hitachi, Japan) after coating with platinum.

**Observation of Gelation Behaviors.** The gelation process of SF solution (4.3% w/w) and GOx (0.05% w/w) blended solution was monitored through turbidity changes (absorbance at 550 nm, 25°C) with a microplate reader (SpectraMax M2, Molecular Devices, USA).

**Characterization of Secondary Structures.** The secondary structures of lyophilized nanoreactors were determined by a Fourier transform infrared (FTIR) spectrometer (Nicolet iZ10, Thermo Scientific, USA) equipped with a Smart iTR^TM^ attenuated total reflection (ATR) diamond crystal cell (Thermo Scientific, USA) in reflection mode. For each measurement, 64 scans were coded at a resolution of 4 cm^–1^, with the wavenumber ranging from 400 to 4000 cm^–1^. Fourier self-deconvolution (FSD) of the infrared spectra covering the amide I region (1585–1725 cm^−1^) was performed using Peakfit v4.12 software to analyze the secondary structures of protein samples. An X-ray diffractometer (Bruker D8 Advance, Germany) was also used to characterize the crystalline structures of protein samples. 2 theta was ranged from 5 to 50 degree, and the scanning speed was 2 s per step while the step size was 0.01 degree.

**Measurement of Enzyme Activity.** Enzyme activities of SENs (GOX) and SENs (GOx-HRP) were accessed by employing the glucose as the substrate and monitoring the oxidation rate of ODS. Briefly, a phosphate buffer (0.1 м, pH = 7.0) containing 2 × 10^−4^ м ODS, SENs (enzymes/SF mass ratio: 1/240) or free enzymes with given concentrations and a specific amount of substrate (glucose), were incubated at 25˚C for 5 min. During the incubation, absorbance at 460 nm was recorded continuously with a microplate reader (SpectraMax M2, USA). The absorbance curve was plotted versus time and ΔA_460_ from the linear portion of the curve was calculated. The activity assays were repeated with a series of appropriate concentrations of substrate to get a series of catalytic rates to obtain $K_{M}^{\mathrm{app}}$ by the Lineweaver–Burk plot. Similar assays were also performed with free enzymes mixture (free GOx+HRP) as the control experiment, which contained the same amount of enzymes (enzyme molar ratio: 1) as SENs (GOx-HRP). The Michaelis constant ($K_{M}^{\mathrm{app}}$) is equal to the substrate concentration at which the reaction rate is half of the maxium rate of the system. A detailed example for determining the kinetic parameter is presented in Figure S3 and Table S1.

**Stabilities and Protease Resistance Test.** Stabilities of both free enzymes and SENs under polar organic solvent were tested by determining the residual activities of free HRP mixture (HRP: 0.025 μg ml^−1^) and SENs containing the same amount HRP (enzyme/SF mass ratio: 1/30, 1/60, 1/120, 1/240, 1/480, 1/960) incubated in the acetone at room temperature (RT). The residual activities of HRP were accessed by employing the hydrogen peroxide (H_2_O_2_) as the substrate and monitoring the oxidation rate of TMB.^[36]^

Thermal stability of both free enzymes and SENs were tested by determining the residual activities of 0.1 μg ml^−1^ GOx and SENs containing the same amount GOx (enzyme/SF mass ratio: 1/240) after incubating in 60°C aqueous solution for several periods. The residual activities of GOx were accessed by employing the glucose as the substrate and monitoring the oxidation rate of TMB under the catalysis of HRP.

Storage stabilities of both free enzymes and SENs were tested by determining the residual activities of 0.01 μg ml^−1^ HRP and SENs containing the same amount HRP (enzyme/SF mass ratio: 1/240) after incubating in aqueous solution at RT for several periods. The residual activities of HRP were accessed by employing the hydrogen peroxide as the substrate and monitoring the oxidation rate of TMB.^[36]^

Stabilities of both free enzymes and SENs under viscous environments were tested by determining the residual activities of free GOx/HRP mixture (GOx: 0.1 μg ml^−1^, HRP: 0.025 μg ml^−1^) and SENs containing the same amount GOx/HRP (enzyme/SF mass ratio: 1/240) in the polyethylene glycol (PEG, M_W_ ≈ 3000) solutions with a series of PEG concentrations (0%, 5%, 10%, 15%, 25% and 30% m/v). The residual activities were accessed by employing the glucose as the substrate and monitoring the oxidation rate of ODS.

Protease resistance of both free enzymes and SENs were tested by determining the residual activities of 0.025 μg ml^−1^ AOx and SENs containing the same amount AOx (enzyme/SF mass ratio: 1/240) after incubating in simulated intestinal fluid (PH = 7.6, 0.1 м phosphate buffer) containing 1 × 10^−5^ м trypsin (≥10,000 BAEE units/mg) at 37°C for several periods. The residual activities of AOx were accessed by employing the alcohol as the substrate and monitoring the oxidation rate of TMB under the catalysis of HRP.

**Efficiency of H_2_O_2_ and Alcohol Elimination.** SF-GOx nanoreactors or SF-GOx/Cat (enzymes mass ratio: 1) nanoreactors were added into 0.01 м glucose solution (PH = 4.5, 0.05 м PBS) with 5×10^−4^ м TMB and 1 μg mL^−1^ HRP. The final concentration of GOx was 0.4 μg mL^−1^. The reaction mixtures were vortexed and subsequently transferred into a 96-well plate. During the reaction at RT, absorbance at 650 nm was recorded continuously with a microplate reader (SpectraMax M2, USA). Similar procedures were further performed with free GOx and GOx/Cat (concentration of GOx and Cat: 0.125 μg mL^−1^) as control experiments. In order to relate the concentration of H_2_O_2_ with the absorbance, a standard absorbance cure V.S. H_2_O_2_ concentration was obtained by the following procedure. H_2_O_2_ solutions with a series of concentrations were added into 0.01 м glucose solutions (pH = 4.5, 0.05 м PBS) with 5 × 10^−4^ м TMB and 1 μg mL^−1^ HRP, respectively. The final concentrations of H_2_O_2_ were 5, 10, 20, 40, 60 and 120 μм. The mixtures were vortexed and the reactions were conducted for 30 min at RT to allow the complete conversion of H_2_O_2._ Then, the mixtures were transferred into a 96-well plate and the absorbance at 650 nm was recorded by the microplate reader. The absorbance of samples was plotted V.S. concentration of H_2_O_2._

The eliminating efficiency of alcohol was detected by following experiments. AOx/Cat mixed free enzymes (enzyme molar ratio: 1) or SENs containing the same amount of enzymes were added into simulated intestinal fluid, which contains 2 × 10^−5^ м alcohol (the final concentration of AOx is 2 μg ml^−1^). After reacting for several periods (0, 0.5, 1, 2, 3, 5 and 6 h) at 37°C, the residuals of alcohol were measured using an ethanol assay kit (BioVision, USA).

**Cell Viability Assays.** (1) 3T3 cells were treated with indicated doses of ethanol/SNPs for 4h. (2) 3T3 cells were treated with PBS, SNPs (final concentration 40 μg ml^−1^) or SENs containing AOx and Cat (final concentration SF: 40 μg ml^−1^, AOx: 2 μg ml^−1^, Cat: 0.5 μg ml^−1^) together with ethanol (final concentration 4%) for 4h at 37°C. Cell viability was measured using MTT assay cell viability rates were normalized with those of the untreated cells. Cell viability was measured using (3-[4,5-Dimethylthiazol-2-yl]-2,5-diphenyltetrazolium bromide (MTT) assay.

***In-Vivo* Studies.** All *in vivo* experimental procedures were operated under animal care protocols approved by Xiamen University Animal Care and Use Committee.

Male C57B6 mice (8 weeks old) from Xiamen University Laboratory Animal Center were used in these studies. 16 mice were divided into 4 groups and fasted for 12 h before the studies. 3 groups were gavaged with alcohol diets (2mg ethanol per gram bodyweight) containing PBS, SNPs, and SENs containing AOx and Cat, respectively. The doses of SENs were maintained at 300 μg SF, 60 μg AOx and 30 μg Cat per mouse, and the volume of PBS or SNPs or SENs solutions was maintained at 300 μl per mouse. Blank group of mouse were fed with isocaloric diets without ethanol. Blood samples (4 μl) were taken from tails at 0.5 h, 1 h, 2 h and 4 h after alcohol feeding, and blood alcohol concentrations were determined using an ethanol assay kit (BioVision, USA). The levels of serum ALT were analyzed (10 μl serum per sample) 6–7 h after alcohol gavage using a mouse ALT ELISA kit (R&D systems, USA).

**Statistical Analysis.** To determine the statistical significance of observed differences between the study groups, a two-tailed homoscedastic *t*-test was applied. Data are presented as mean±standard error of the mean (s.e.m.) and the significance levels are **P* < 0.05, ***P* < 0.01 and ****P* < 0.001. P < 0.05 or less was considered significant.


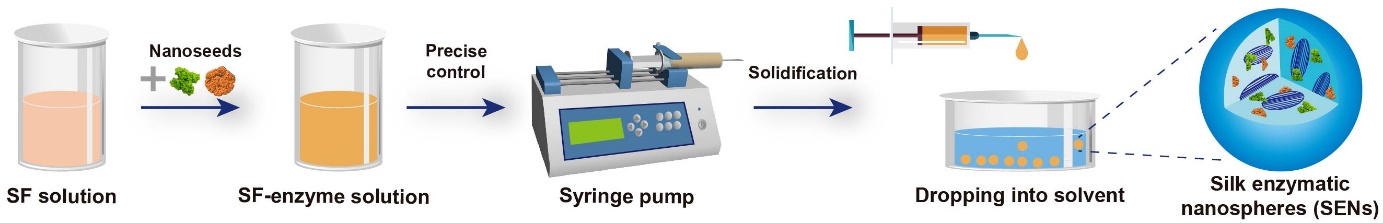


**Scheme S1.** Schematic of the procedures for obtaining silk enzymatic nanospheres (SENs). i) Regenerated silk fibroin (SF) solution was prepared using the traditional methods; ii) selected enzymes were added in regenerated SF solution; iii) the SF-enzyme solution was gently introduced into coagulant through a syringe pump and a sample injector (~150 μL drop^−1^); iv) SENs were obtained through a purification process (***Supplementary Information***).


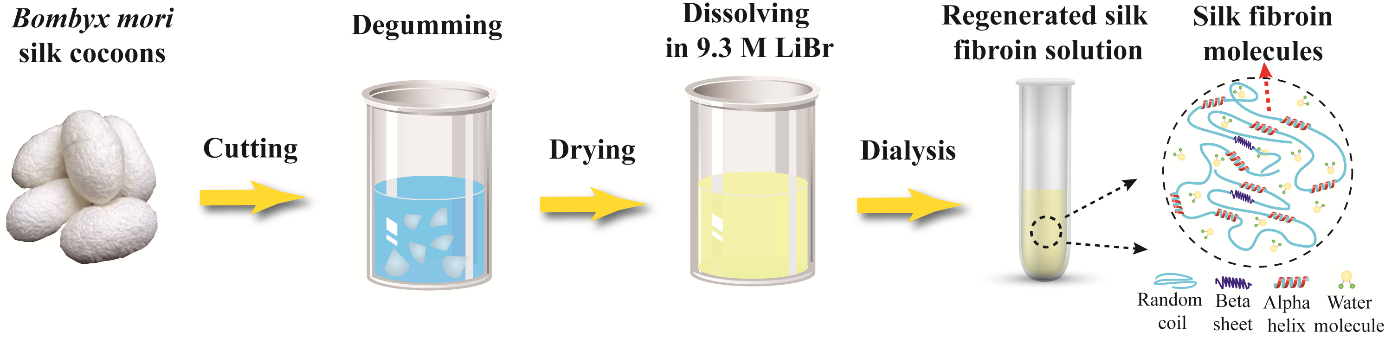


**Scheme S2.** Preparation process of regenerated SF solution.


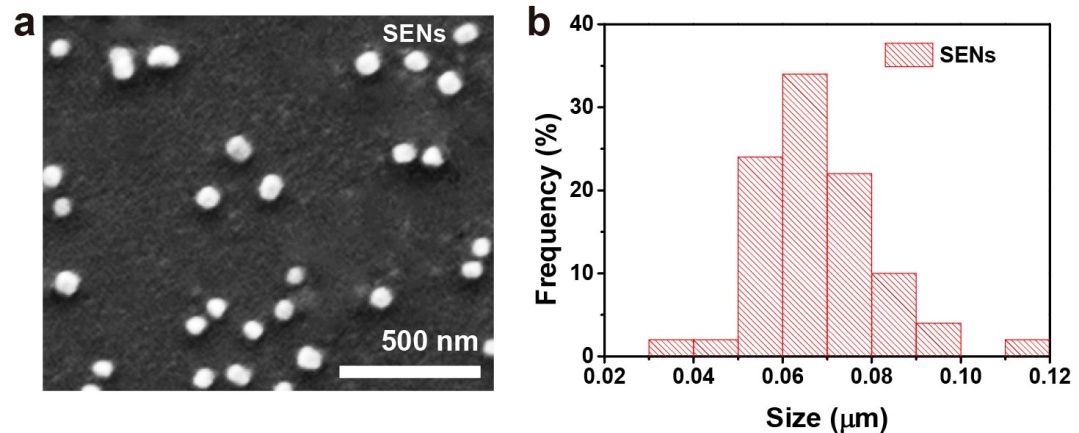


**Figure S1.** SEM images of SENs; scaling bars are 500 nm.


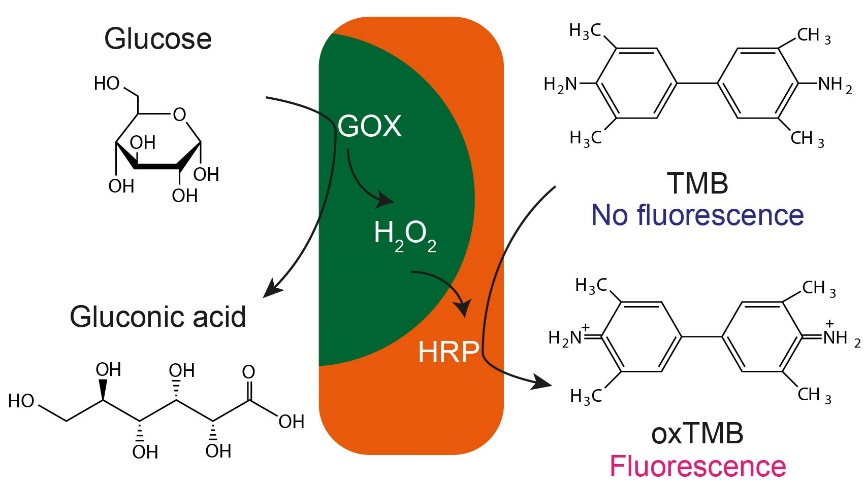


**Figure S2.** Schematic illustration and reaction equations of GOX-HRP-catalyzed TMB oxidation for the production of oxTMB after initiation by glucose.


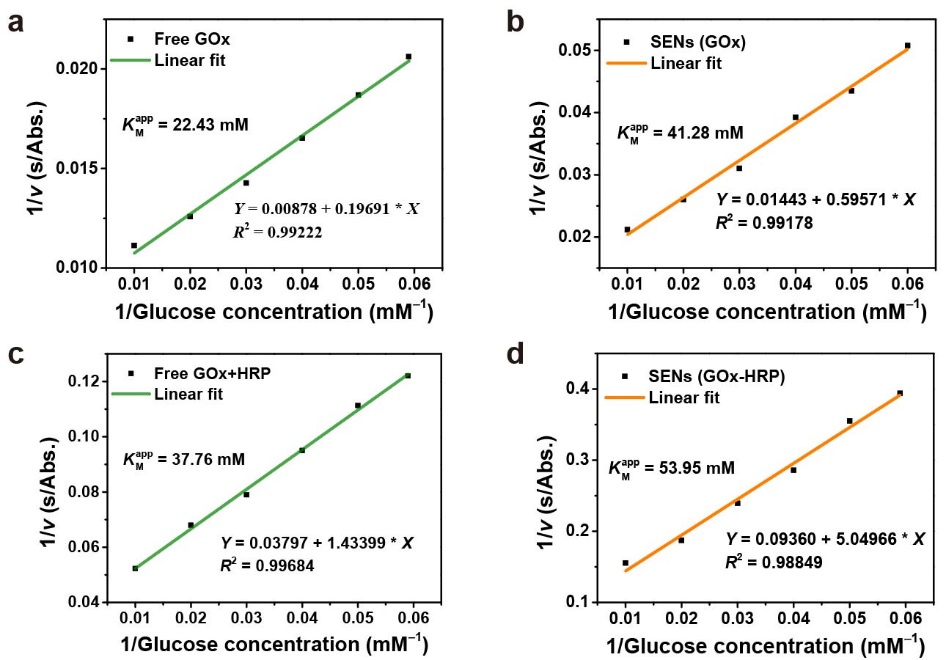


**Figure S3.** Lineweaver-Burk plots for comparison of Michaelis-Menten parameters (*K*m) of a) free GOx, b) SENs (GOx), c) free GOx+HRP (enzyme molar ratio: 1) and d) SENs (GOx-HRP) (enzyme molar ratio: 1).


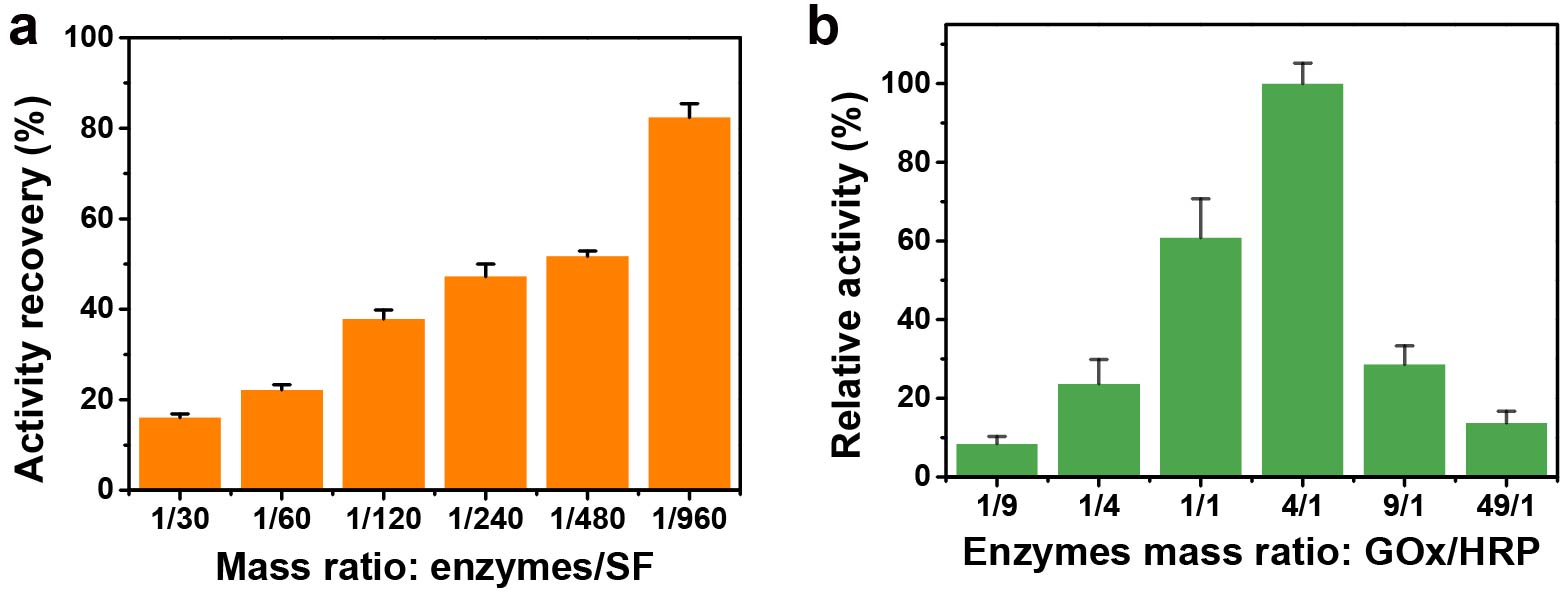


**Figure S4.** (a) Recoveries of enzymes’ activities of SENs with various enzymes/SF mass ratios. (b) Relative activities of SENs with various molar ratios of enzyme GOx and HRP.


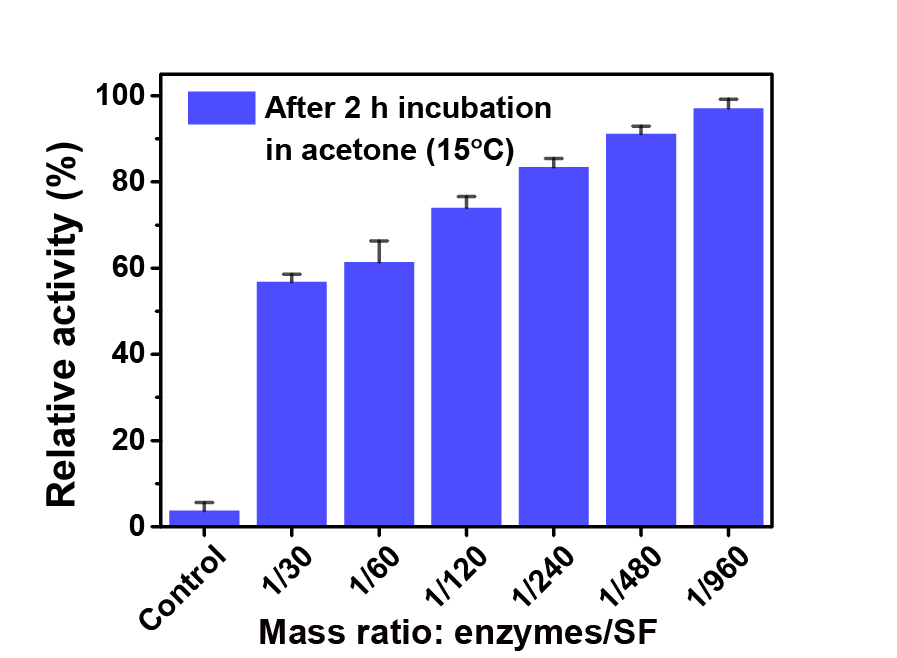


**Figure S5.** Relative activities of free enzymes (HRP) and SENs with various enzyme/SF mass ratios after 2 h incubation in acetone at 15°C.


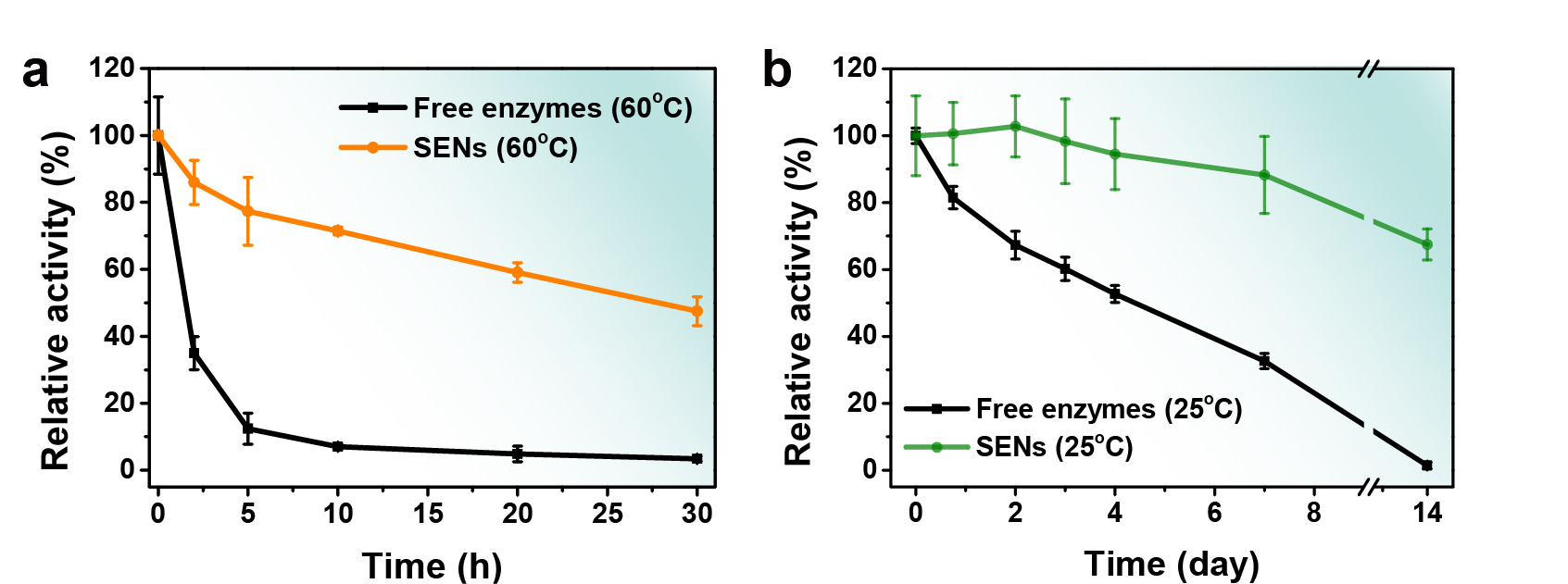


**Figure S6.** Stabilities of free enzymes and SENs incubated in aqueous solution (a) at 60°C and (b) at 25°C.


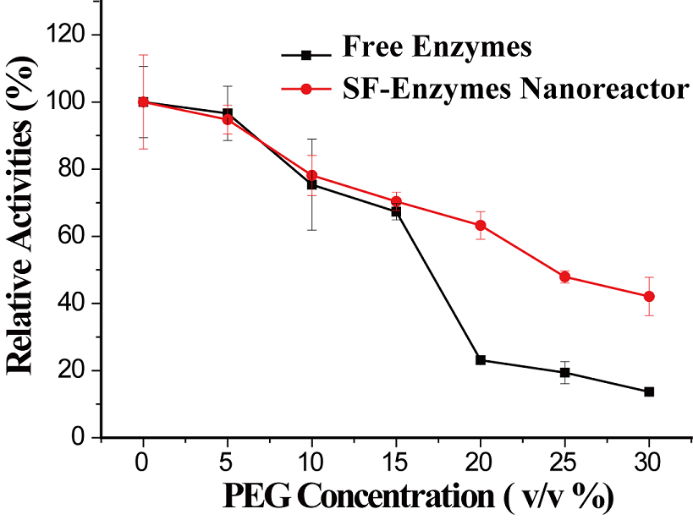


**Figure S7.** Relative activities of free enzymes (GOx/HRP) and SENs containing the same amount enzymes in the presence of increasing concentrations of PEG in phosphate buffer (50 mM, pH 7.0).


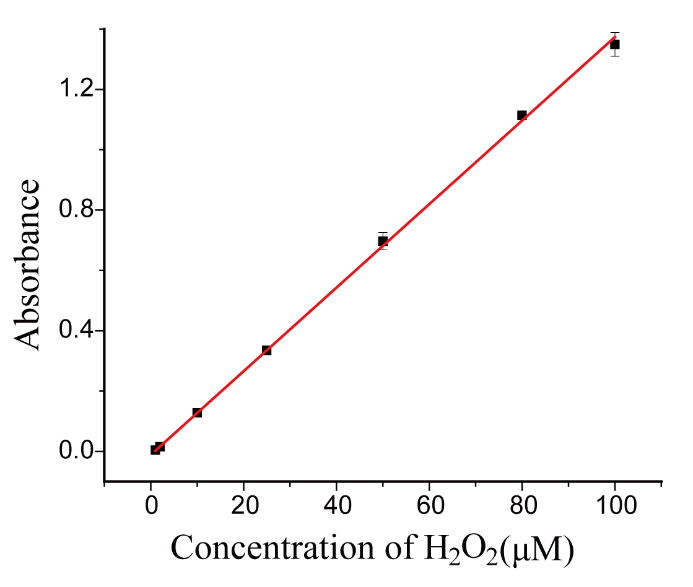


**Figure S8.** Standard curve represents the relationship between absorbance and H_2_O_2_ concentration. Data represent average; errors come from three independent experiments performing triplicated.


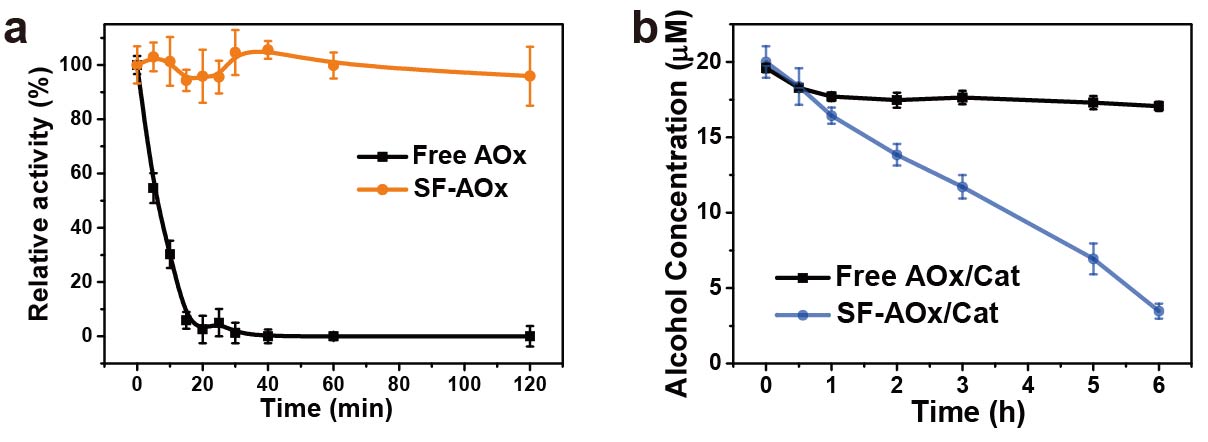


**Figure S9.** a) Relative activities of free enzymes (AOx) and SENs containing the same amount enzymes incubated in the simulated intestinal fluid. b) Residuals of alcohol in the simulated intestinal fluid after catalysis reaction of free enzymes (AOx/Cat, enzyme molar ratio: 1) or SENs containing the same amount enzymes.


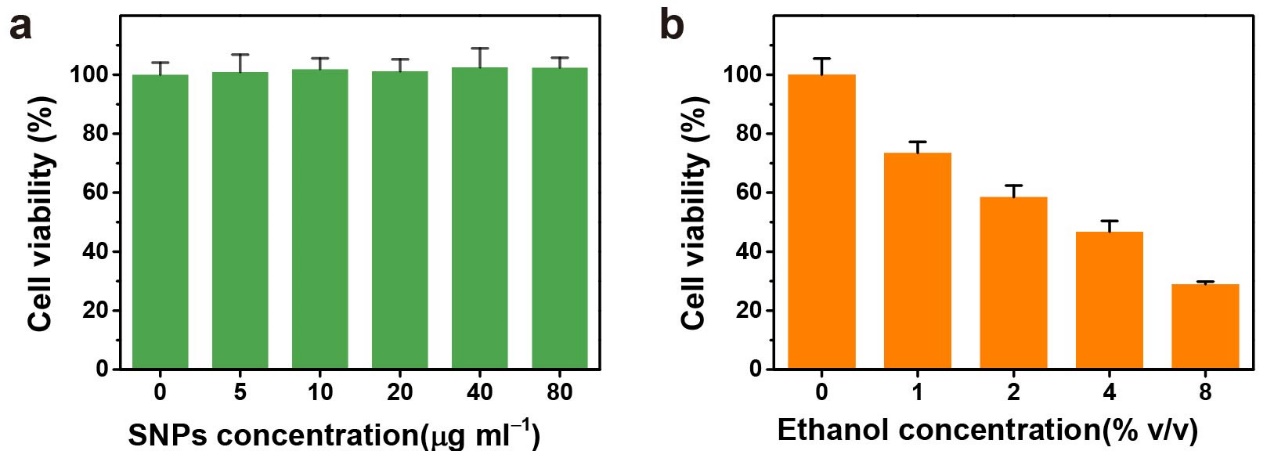


**Figure S10.** a) b) Cell viability assays after treated with indicated doses of SNPs for 4 h. Cell viability rates were normalized with those of the untreated cells. Cell viability was measured using MTT assay.


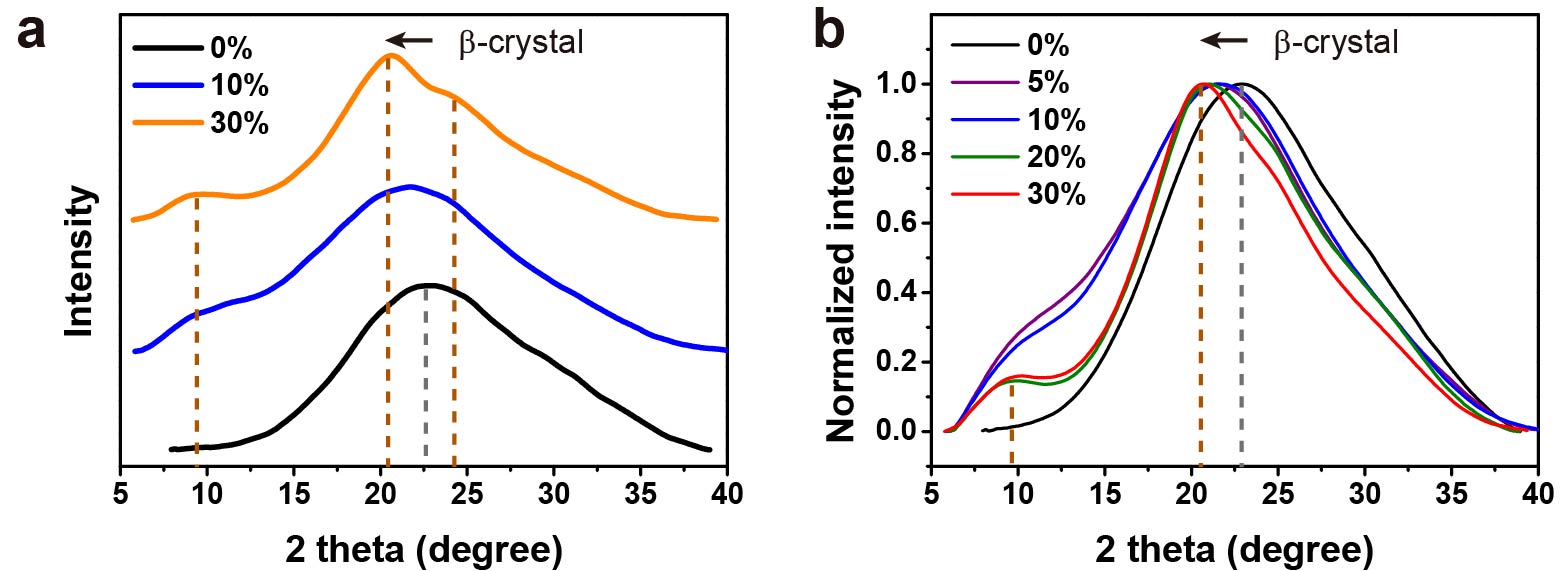


**Figure S11.** XRD spectra of GOx/SF samples at various GOx/SF ratios (0, 5, 10, 20 and 30 wt %, with the concentration of SF kept at 5 wt %, after ~60 h of incubation at *T* = 25°C).

**Table S1.** Kinetic data for Kinetic data for SENs (GOX-HRP) and free GOX and HRP.

| **System** | **Substrate** | **Others** | $\boldsymbol{K}_{\mathbf{M}}^{\mathbf{app}}$ **(× 10^−3^ м)** |
| --- | --- | --- | --- |
| Free GOx | Glucose | HRP, ODS | 22.43 |
| SENs (GOx) | Glucose | HRP, ODS | 41.28 |
| Free GOx+HRP | Glucose | ODS | 37.76 |
| SENs (GOx-HRP) | Glucose | ODS | 53.95 |
